# Endophytic bacteria and fungi as a source of polysaccharides: production and characterization techniques

**DOI:** 10.3389/fmicb.2026.1903754

**Published:** 2026-09-14

**Authors:** Meera Mathew, Mythili Sathiavelu

**Affiliations:** Department of Biotechnology, School of Biosciences and Technology, Vellore Institute of Technology, Vellore, Tamil Nadu, India

**Keywords:** characterization, endophytic bacteria, endophytic fungi, polysaccharides, production

## Abstract

Polymers, mainly polysaccharides, are a class of macromolecules that have a variety of structural and functional roles in biological systems. Recently, research on polysaccharides, especially exopolysaccharides (EPS) from microbial sources, has gained significant interest. These substances are valued for their biocompatibility, biodegradability, and wide range of applications across the pharmaceutical, biomedical, and industrial sectors. Endophytic microorganisms, which live inside healthy plant tissues, are a newly discovered sustainable source of exopolysaccharides. This article reviews research on endophytic bacteria and fungi that produce polysaccharides. The major focus is on summarizing the production and characterization techniques used. It includes extraction, purification, and structural analysis, including chromatographic, spectroscopic, and microscopic techniques. Additionally, tables summarizing features of polysaccharides from both bacterial and fungal endophytes, and a table on the characterization techniques and biological activities of these polysaccharides are included. By consolidating current insights and identifying existing gaps, this review seeks to enhance understanding of endophytic polysaccharides.

## 1 Introduction

Polymers consist of large molecules formed from multiple repeating units of smaller molecules known as monomers. It consists of a broad class of both natural and synthetic macromolecules with diverse origins and functionalities (Huang et al., 2024). Among these, polysaccharides represent a significant subgroup of carbohydrate-based polymers. Polysaccharides are large molecular weight (MW) carbohydrates formed through polymerization of more than 10 monosaccharide units (Yadav et al., 2024; Mohammed et al., 2021; Ding et al., 2025). It is distributed in all living organisms; they serve as key energy storage molecules and as structural components of cell membranes and cell walls, thereby playing vital roles in physiological processes such as signal transduction and osmotic regulation (Gong et al., 2022). In addition, polysaccharides show a wide range of biological activities, including antitumor, immunomodulatory, anti-inflammatory, antibacterial, antioxidant, and antiviral activities, which underpin their growing importance in biomedical and industrial applications (Li et al., 2026; Chen and Huang, 2018; Wu et al., 2022; Yu et al., 2019). Within the category of polysaccharides, microbial polysaccharides comprise a distinct and extremely useful group due to their unique metabolic capabilities, which enable the production of structurally complex and functionally diverse macromolecules (Gul et al., 2025).

There are both synthetic and natural forms of polysaccharides. Natural polysaccharides, or biopolysaccharides, are widely distributed in living systems and are primarily derived from plants, animals, microbes, and algae (Figure 1) (Chen et al., 2021). Bio-polysaccharides are necessary for living organisms. They contribute to both structural stability (as components of cell walls and membranes) and cellular composition, as intracellular polysaccharides (IPS) found in the cytoplasm (Shi, 2016).

**Figure 1:**
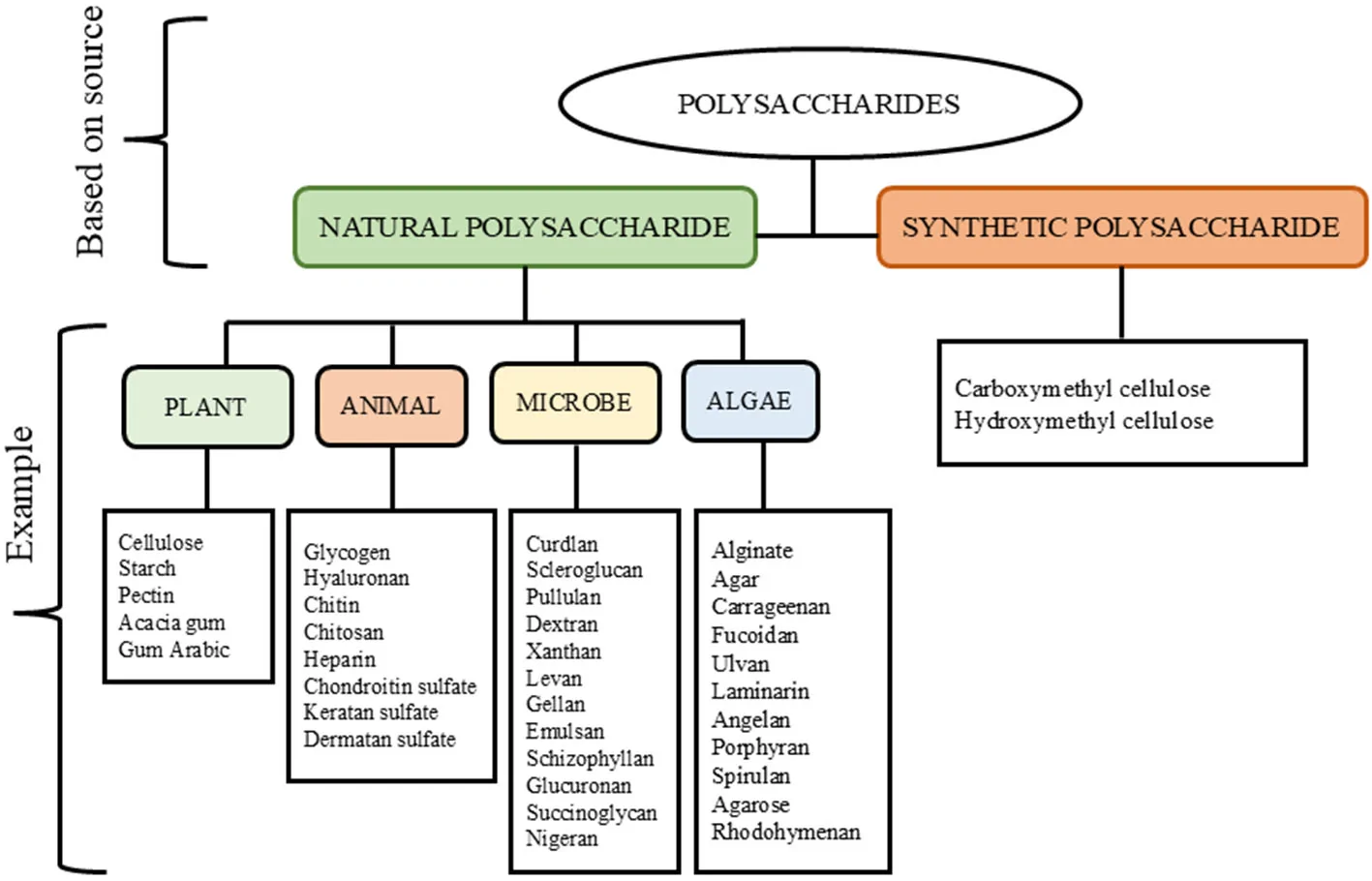
Classification of polysaccharides based on source and origin.

Among these, microbial polysaccharides have gained popularity in recent decades due to ease of large-scale production under controlled fermentation conditions, considerably shorter production times, ease of extraction, structural diversity, biocompatibility and biodegradability. This makes them highly valuable in the biomedical, pharmaceutical, food, cosmetic, and industrial sectors (Figure 2) (Song et al., 2023). In the food industry, these are widely used as thickening agents, emulsifiers, stabilizers, moisture retention agents and gelling agents to enhance texture, shelf life, thickness and quality (e.g., xanthan gum, dextran, curdlan, gellan gum) (Subhash et al., 2026; Jindal and Singh Khattar, 2018; Natarajan et al., 2022). In the cosmetic industry, these microbial polysaccharides are used as moisturizers, film-formers, and stabilizers in formulations such as creams, lotions, shampoos, and toothpaste due to their water-binding property and biocompatibility. In the biomedical and pharmaceutical field, these microbial polysaccharides are used as drug carriers, wound dressings, tissue engineering scaffolds, microcarriers for cell culture, and bioactive substances with antioxidant, antimicrobial, immunomodulatory, anticancer, cholesterol-lowering, antiviral, and prebiotic properties (e.g., hyaluronic acid, pullulan, levan). Additionally, microbial polysaccharides are used in diverse fields such as agriculture, textiles, oil recovery, environmental protection, and packaging, and they are versatile, sustainable, and high-value biopolymers across multiple industrial sectors (Yildiz and Karatas, 2018).

**Figure 2:**
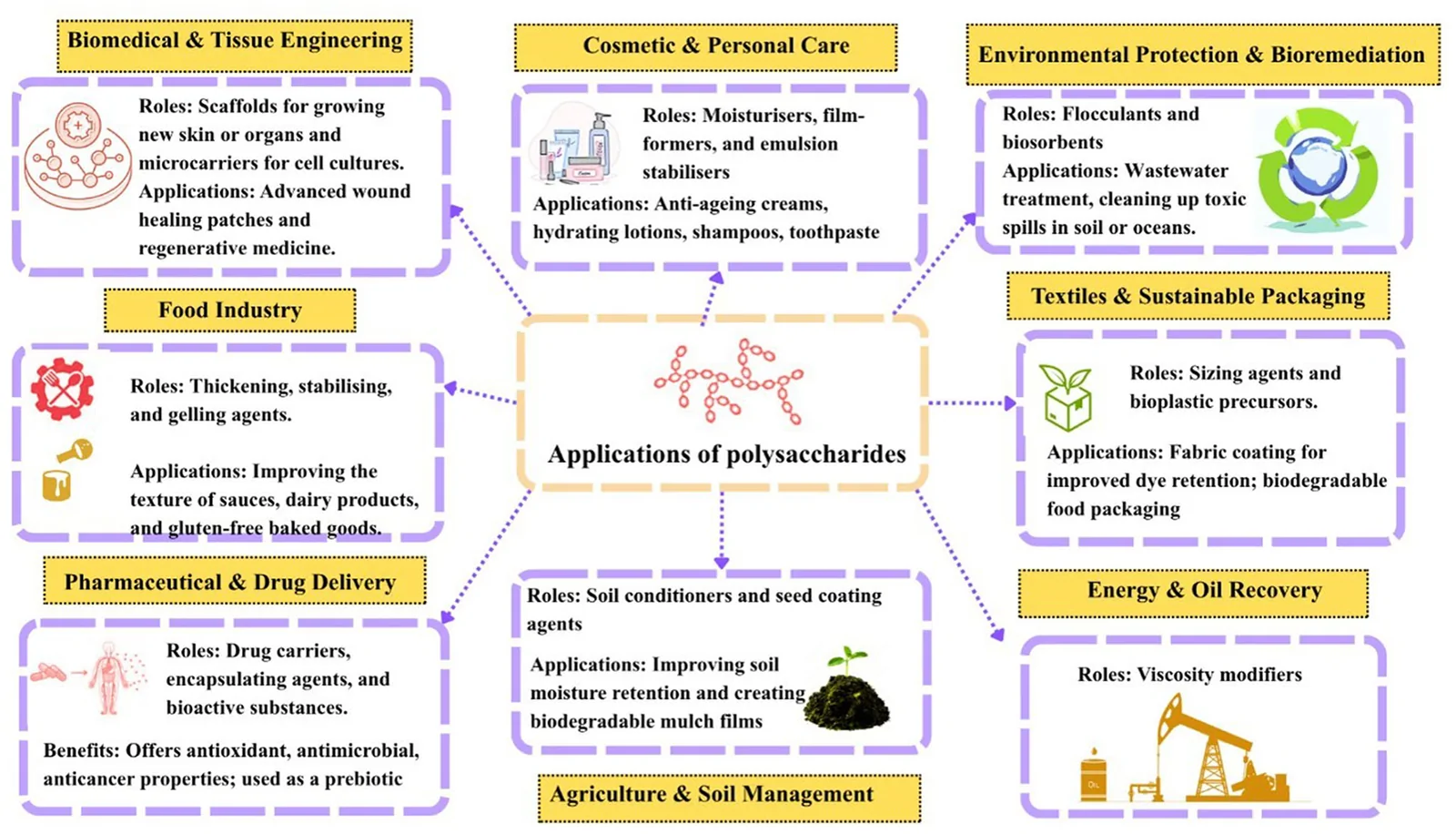
Schematic representation of the various applications of polysaccharides.

Recently, there has been increasing interest toward plant-associated microorganisms as a novel alternative and sustainable source of polysaccharides (Burragoni and Jeon, 2021). These microorganisms are called endophytes, which live inside healthy plant tissues while not harming the host plant and forming long-term symbiotic relationships with their hosts. The intimate relationship between the host and the microbe creates unique physiological and chemical pressures that shape endophyte metabolism, leading to the production of polysaccharides with distinct structural and functional properties (Raimi and Adeleke, 2021). Consequently, polysaccharides from endophytes, particularly EPS, are increasingly recognized as valuable biomolecules with high potential for biomedical applications, thereby establishing endophytic microorganisms as a new and unexplored area in microbial polysaccharide research (EL-khawaga et al., 2024; Gul et al., 2025).

This review article gives an overall summary of the literature on exopolysaccharide-producing endophytic microorganisms, focusing on their host plant sources, production characteristics, chemical composition, and structural diversity. Furthermore, the review explains the biological functions of these polysaccharides. This review also explains existing knowledge gaps and future research directions in this emerging and underexplored field.

## 2 Literature collection strategy for review

The literature included in this current review was searched from online databases such as Scopus, Web of Science, PubMed, and Google Scholar, and the reference lists of relevant articles were also screened to identify additional studies. This review primarily includes recent research articles describing the production, extraction, purification, structural characterization, and biological activities of polysaccharides isolated from endophytic microorganisms. Some review articles were also considered to provide background information. Priority was given to research works which is published between 2019 and 2026, with particular emphasis on publications from 2023 to 2025. Although the search strategy applied for bacterial and fungal endophytes was the same, a greater number of studies on this topic were available on fungal endophytes. Therefore, fungal examples are more extensively represented in this review. This disparity highlights the need for more research on bacterial endophyte-derived polysaccharides.

## 3 Types of microbial polysaccharides

Microbial polysaccharides can be generally categorized based on their location of origin into intracellular polysaccharides (IPS), extracellular polysaccharides (exopolysaccharides, EPS), and cell wall polysaccharides (CWPS) (Khan et al., 2022) (Figure 3). EPS are produced in the cell and secreted into the extracellular space, often as a high molecular weight homo or heteropolymer, and have been the most well-studied polysaccharides because of their ease of isolation. IPS, on the other hand, remain in the cytoplasm or cellular structures and are usually isolated from fungal mycelia or fruiting bodies, although their isolation is more difficult; however, this has hindered extensive studies on these polysaccharides. CWPS are an essential component of the microbial cell wall, and while they may partially overlap with IPS, they are an important part of the microbial cell wall and play a role in cell wall integrity and protection.

**Figure 3:**
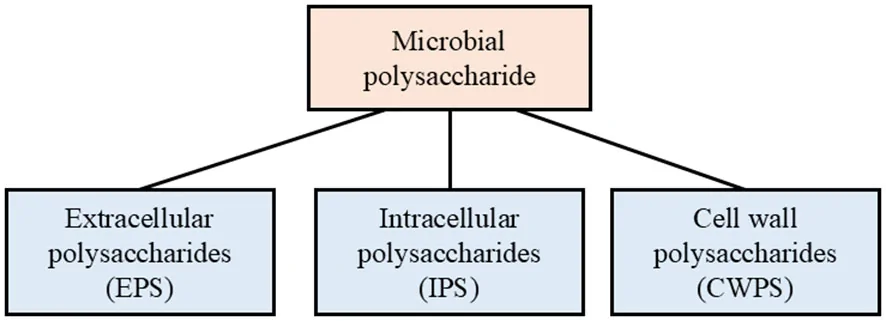
Types of microbial polysaccharides based on cellular localization.

## 4 Plant endophytes as a source of structurally diverse bioactive compounds

Endophytes are microorganisms that reside within the healthy plant tissues without causing any apparent disease symptoms (Nair and Padmavathy, 2014). Through long-term coevolution, these microorganisms form mutualistic associations with their host plants, often enhancing host defense mechanisms, stress tolerance, nutrient acquisition and other benefits to the plant. In return, plants provide a protected ecological niche that supports endophytic survival and metabolic activity (dos Santos, 2025). Endophytes can produce a wide variety of bioactive compounds similar to or even more potent than those produced by their host plant. This synthesis capability makes endophytes ecologically sustainable and renewable sources of valuable natural products (Wen et al., 2022).

Endophytes are of different types. They consist of bacteria, actinomycetes, mycoplasmas, and fungi (Golinska et al., 2015). These microbes form mutualistic or symbiotic interactions with plants, making substantial contributions to plant development, stress tolerance, and defense mechanisms (Ullah et al., 2025). Over 200 genera from 16 different phyla of bacterial species have been identified as endophytes, with the majority of the species belonging to actinobacteria, proteobacteria, and firmicutes phyla (Vandana et al., 2021). Bacterial endophytes exhibit a wide range of diversity and are known to generate unique bioactive metabolites that function as antibacterial, anti-inflammatory, anticancer, etc. (Choudhury et al., 2022).

Actinomycetes are prokaryotic microorganisms from the Actinobacteria phylum that have fungus-like mycelium, and they produce spores (Silva et al., 2022). Historically, actinomycetes were regarded as intermediate forms between fungi and bacteria (Barka et al., 2015). These endophytic actinomycetes are also recognized for producing a variety of chemical entities with distinct structures that have significant medical value (Anavadiya et al., 2024). Mycoplasma species are also reported as plant endophytes (Islam et al., 2025).

Endophytic fungi comprise a varied collection of microorganisms that typically exist without causing symptoms in plant tissues or the spaces between cells. They encourage the growth of host plants by generating secondary metabolites that boost the plant's resilience to abiotic and biotic stress factors (Akram et al., 2023). In addition, they possess the ability to biosynthesise therapeutically important phytochemicals that were thought to be exclusively generated by the host plant (Tiwari and Bae, 2022). Endophytic fungi belong mainly in the phyla *Ascomycota and Basidiomycota. Penicillium, Fusarium, Pestalotiopsis*, and *Taxomyces* are common genera found in a variety of plant hosts (Gouda et al., 2016).

## 5 Endophyte-derived polysaccharides: diversity, structural characteristics and biological significance

Endophytic microbes are emerging as promising producers of structurally varied exopolysaccharides (EPS) (dos Santos, 2025). Bacterial and fungal endophytes produce high-molecular-weight carbohydrate polymers with distinct compositions, molecular structures, and functional characteristics. Tables 1, 2 highlight the polysaccharides' variety, structural characteristics, and biological relevance.

**Table 1:** Endophytic bacterial strains and their reported polysaccharides, production characteristics, structural features, and biological activities.

No:	Endophytic bacteria	Host plant	Type of polysaccharide	Production yield	Monosaccharide composition	Glycosidic linkage	Molecular weight	Biological activity	References
1	*Burkholderia* sp.	*Grifola frondosa*	EPS	11.36 g/L	Gal: Glc: Man = 39.52: 14.22: 46.26	[→4)-α-D-Glcp-(1→6)-β-D-Galp-(1→]_n_	432.05 kDa	Antibacterial activity, prebiotic activity	Xie et al., 2025
2	*Methylorubrum* sp.	*Taxus cuspidata* cv. Nana	EPS	1.36 mg/ml	Man: Rha: GlcA: GalA: Glc: Gal: Xyl: Ara = 21.83: 3.12: 5.58: 5.09: 26.87: 9.98: 1: 3.92	β-glycosidic linkages	12.6 kDa	Antioxidant	Liang et al., 2025
3	*Methylorubrum* sp.	*Tremella aurantialba*	–	6.94 g/L	Ara: GlcN: Gal: Glc: Man = 0.073: 0.145: 0.406: 0.182: 0.195	[→4)-β-D-Galp-(1→4)-β-D-Manp-(1→]_n_	5.408 kDa	Probiotic activity	Fei et al., 2024
4	*Lysinibacillus sphaericus*	*Ginkgo biloba*	EPS	10.265 g/L	Rha: Ara: Fuc: Xyl: Man: Glc: Gal = 2.55: 1.10: 1.00: 2.90: 17.03: 6.99: 23.85	–	13.20 kDa	Antioxidant activity, Antibacterial activity	Sun et al., 2024
5.	*Pseudomonas flavus Y11*	*Zanthoxylum bungeanum*	EPS	1.21 g/L	Man: Rha: GlcA: GalA: Glc: Gal: Xyl: Ara: Fuc = 5.5: 0: 13.4: 1.1: 13.2: 42.0: 10.5: 0.9: 13.4	–	26.70 kDa	Antioxidant, antibacterial	Long et al., 2024
6.	*Rathayibacter oskolensis*	*Androsace koso-poljanskii*	CWPS	–	Rha, Man, Xyl	[→3)-α-D-Rhap-(1→2)-α-D-Manp-(1→]_n_	30 kDa	–	Shashkov et al., 2024
7.	*Rathayibacter oskolensis*	*Androsace koso-poljanskii*	CWPS	–	Man, Gal, Glc, Rha	[→4)-α-D-Manp-(1→3)-α-D-Galp-(1→3)-β-D-Glcp-(1→]_n_	–	–	Shashkov et al., 2024
8.	*Pseudomonas otitidis*	*Tribulus terrestris L*	EPS	–	–	–	–	Drug delivery application	Kalimuthu et al., 2023
9.	*Bacillus* sp	*Polygonatum sibiricum*	EPS	1.7 g/L	Gal: Glc: Man = 15: 3: 1	–	18.63 kDa	Antioxidant, antitumor	Zhang et al., 2021

Monosaccharide abbreviations: Gal, galactose; Glc, glucose; Man, mannose; Rha, rhamnose; GlcA, glucuronic acid; GalA, galacturonic acid; Xyl, xylose; Ara, arabinose; Fuc, fucose; GlcN, glucosamine. Glycosidic bond: Glcp, glucopyranose; Galp, galactopyranose; Manp, mannopyranose; Rhap, rhamnopyranose.

**Table 2:** Endophytic fungi and their reported polysaccharides, structural characteristics, and biological activities.

No	Endophyte	Host plant/source	Type of polysaccharide	Production yield	Monosaccharide composition	Glycosidic bond	Molecular weight	Biological activity	References
1	*Talaromyces* Sp.	Wheat bran	EPS	3.34 ± 0.17 g/L	Ara: Gal: Glc: Man: GlcA = 0.0985: 4.0208: 35.6949: 2.0528: 0.1718	β-(1→3)-D-glucopyranosidic linkages (dominant), with β-linked heterosaccharide branches	235.187 kDa	Anti-inflammatory Activity	Wang et al., 2025
2	*Ovatospora brasiliensis*	*Pogonatum inflexum* (moss)	EPS	–	Gal, Glc, Man, GlcUA	–	Average molar mass = 100–1,000 kDa	Anti-inflammatory activity, cell–surface interaction capability, and suitability for biocompatible material	Yang et al., 2025
3	*Diaporthe sp*	*Cinnamomum burmannii*	EPS	3.12 ± 0.26 mg/ml	Glc, Man, Gal	1,4-Glcp	462 kDa	Moisture-retentive, antioxidant, anti-inflammatory, hepatoprotective	Zhang et al., 2025
						1,6-Glcp			
4	*Schizophyllum commune*	*Basidiomycete fungus*	EPS	5.10 ± 2.00 g/L	Glc	–	2.5–5,000 kDa (strain-dependent)	Antitumor, immunostimulatory, gel-forming	Prathumpai et al., 2025
5	*Aspergillus fumigatus*	*Bruguiera gymnorhiza*	EPS	2.13 ± 0.38 g/L	Gal, Glc	–	–	Antioxidant, antibacterial	Nyaisaba et al., 2025
6	*Preussia isabellae*	*Bruguiera gymnorhiza*	EPS	1.04 ± 0.17 g/L	Gal, Glc, Fru, Xyl	–	–	Antioxidant, antibacterial	Nyaisaba et al., 2025
7	*Monascus purpureus*	*Cyclocarya paliurus*	EPS	31.66 ± 0.44 mg/ml	Fuc: Gal: Glc: Man: GalA: GlcA = 1.00: 8.52: 19.61: 15.74: 1.00: 11.84	–	320 kDa	Antioxidant property, cell proliferation activity	Tao et al., 2024
8	*Penicillium citrinum*	*Cyclocarya paliurus*	EPS	60.13 ± 1.93 mg/ml	Ara: Gal: Glc: Man: GalA: GlcA = 1.54: 18.40: 4.34: 48.62: 1.00: 18.08	–	158 kDa	Antioxidant activity, strong cell proliferation activity	Tao et al., 2024
9	*Aspergillus versicolor*	*Cyclocarya paliurus*	EPS	89.51 ± 3.31 mg/ml	Ara: Gal: Glc: Man: Xyl: GalA: GlcA = 31.78: 106.36: 203.87: 240.87: 4.95: 1.00: 100.33	–	114 kDa	Antioxidant property, cell proliferation activity	Tao et al., 2024
10	*Neopestalotiopsis clavispora*	Avocado fruits	EPS	7.86 g/L	–	–	–	Antioxidant activity, biodiesel production	Koutb et al., 2024
11	*Chaetomium globosum*	–	EPS	1.53 ± 0.27 g/L	Glc: Man: Rha: GlcN: Gal: Fru: GlcA = 30.38: 32.22: 9.68: 1.34: 1.59: 0.62: 3.73	–	26.64 kDa	Antioxidant	Wang et al., 2024
12	*Talaromyces purpureogenus*	*Pinus densiflora*	EPS	–	Man: Rib: Glc: Gal = 38.70: 25.02: 19.34: 16.94	(→2)-α-L-Araf-(1→2)-α-D-Galp-(1→3)-α-L-Glcp-(1→3,6)-α-L-Manp-(1→)	5 kDa	Antioxidant, wound healing	Hu et al., 2023
13	*Penicillium janthinellum*	*Acanthus ilicifolius*	EPS	–	Man: Gal = 52.71: 47.29	(→2)-α-D-Manp-(1→), (→4)-α-D-Manp-(1→), (→3)-β-D-Galf-(1→), and (→2)-β-D-Galf-(1→) residues, with partial glycosylation at the C-3 position of the (→2)-β-D-Galf-(1→) residue	10.24 kDa	Anti-diabetic activity	Shao et al., 2023
14	*Annulohypoxylon thailandicum*	*Elaeis guineensis Jacq*.	EPS	0.5 g/L	–	–	–	–	Yurnaliza et al., 2021
15	*Pilidiella guizhouensis*	*Eupatorium chinense* L	EPS	1.67 ± 0.22 g/L	Glc	–	13.4 kDa	Antioxidant activity	Zhang et al., 2020
16	*Alternaria tenuissima*	*Angelica sinensis*	EPS	2.1113 mg/ml	D-GalA: Rha: DMan: Glc: D-Gal = 0.45: 3: 02: 3.25: 1.0: 0.95	–	32.46 kDa	Antioxidant activity	Wang et al., 2019
17	*Fusarium solani* DO7	*Dendrobium officinale*	Polysaccharide 1	2.92 ± 0.15 g/L	Gal: Glc: Xyl: Rha = 3.9: 3.4: 1.7: 1.5	α-D-Glcp-(1→), (→3)-β-L-Rhaf-(1→), (→4)-β-D-Xylp-(1→), (→6)-α-D-Glcp-(1→), (→2,6)-α-D-Glcp-(1→), and (→2)-β-D-Galp-(1→)	1.3 kDa	Antibacterial	Zeng et al., 2019
18	*Fusarium solani* DO7	*Dendrobium officinale*	Polysaccharide 2	2.36 ± 0.29 g/L	Gal: Glc: Ara: Rha = 2.9: 3.4: 1.3: 1.7	β-D-Glcp-(1→), (→2)-α-L-Rhaf-(1→), (→3)-α-L-Araf-(1→), (→4)-β-D-Glcp-(1→), (→4,6)-β-D-Glcp-(1→), and (→3)-α-D-Galp-(1→)	174.6 kDa	Antibacterial	Zeng et al., 2019
19	*Fusarium* sp. A14	*Fritillaria unibracteata* Hsiao	EPS (1)	–	Glc: Rha: Gal: Man: Ara: Xyl = 10.00: 0.55: 0.34: 0.31: 0.06: 0.03	α-glycosidic linkages	24 kDa	antioxidant activity	Pan et al., 2019
20	*Fusarium* sp. A14	*Fritillaria unibracteata* Hsiao	EPS (2)	–	Glc: Rha: Gal: Man: Xyl: Ara = 10.00: 0.88: 0.39: 0.16: 0.06: 0.06	α- and β-glycosidic linkages	5 kDa	antioxidant activity and moderate antiproliferative activity against HepG2 cells	Pan et al., 2019

Monosaccharide abbreviations: Gal, galactose; Glc, glucose; Man, mannose; Ara, arabinose; Rha, rhamnose; Xyl, xylose; Fuc, fucose; Fru, fructose; Rib, ribose; GlcA/GlcUA, glucuronic acid; GalA, galacturonic acid; GlcN, glucosamine; Glcp, glucopyranose; Galp, galactopyranose; Galf, galactofuranose; Manp, mannopyranose; Xylp, xylopyranose; Araf, arabinofuranose; Rhaf, rhamnofuranose.

### 5.1 Structural characteristics and biological activities of polysaccharides obtained from endophytic bacteria

Table 1 summarizes the reported endophytic bacterial strains and their associated polysaccharide production characteristics, including host source, polysaccharide type, yield, composition, structural features, and biological activities. Most of the reported bacterial exopolysaccharides (EPS) are heteropolysaccharides made mainly of glucose, galactose, mannose, rhamnose, and uronic acids in different molar ratios (e.g., *Rathayibacter oskolensis, Pseudomonas flavus Y11*). Mannose- and glucose-rich compositions appear often, indicating their structural importance in bacterial EPS. Some species also include less common sugars like fucose and glucuronic acid, which adds to their structural complexity and possible specific functions (e.g., *Methylorubrum* sp., *Pseudomonas flavus* Y11). The molecular weight range of these polysaccharides varies from around 5 kDa to more than 400 kDa, reflecting the structural heterogeneity in these bacterial-derived EPS. The types of glycosidic bonds, branching pattern, conformation, etc. play a significant role in the biological activities of polysaccharides. EPS obtained from *Burkholderia* species have a high molecular weight, ∝-glucan backbone with O-6 branches, and it shows both antibacterial and prebiotic activities. Many bacterial exopolysaccharides are reported to have strong antioxidant activity. Studies suggest that uronic acid content is important for the antioxidant potential of polysaccharides. Higher uronic acid levels are linked to better scavenging abilities for hydroxyl radicals and superoxide anions (Ai et al., 2020) (e.g., *Pseudomonas flavus*). This diversity of biological activities of bacterial polysaccharides demonstrates their multifunctional nature and their importance in the pharmaceutical and nutraceutical industries.

As shown in Table 2, endophytic fungal polysaccharides have notable structural variety and a wider range of molecular weights compared to bacterial counterparts. Fungal EPS mainly consist of glucose, mannose, and galactose, often with uronic acids, rhamnose, xylose, arabinose, or fucose (e.g., *Aureobasidium pullulans, Colletotrichum alatae, Fusarium*). Many fungal species produce glucose-rich polymers, often identified as β-glucans, which play a crucial role in fungal cell walls (e.g., *Chaetomium globosum, Fusarium*). Glycosidic linkage analysis shows that β-(1→6) and β-(1→3) linkages are frequently found in fungal glucans (e.g., *Fusarium solani* DO7, *Talaromyces purpureogenus*). There are also mixed α- and β-configurations in heteropolysaccharides (e.g., *Fusarium solani* DO7). The molecular weight of fungal polysaccharides varies significantly, ranging from as low as 1 to 5 kDa to several thousand kilodaltons. This variation reflects differences in biosynthesis based on strain and in the branching patterns of polymers. Biologically, fungal polysaccharides exhibit a variety of activities, including antioxidant activity, immunostimulatory, anti-inflammatory, antitumor, hepatoprotective activity, wound-healing property, and anti-diabetic effects. Some also have properties that allow them to form materials, such as gels and moisture retention (e.g., EPS of *Porphyridium cruentum*). This suggests they could be useful in biomedical materials and cosmetic products. The variety in their structure and biological functions highlights the biotechnological importance of these fungal endophytic polysaccharides.

## 6 Production, extraction, purification and characterization of endophyte-derived polysaccharides

### 6.1 Production and extraction of endophytic polysaccharides

The general workflow for producing and characterizing endophyte-derived exopolysaccharides (EPS) is shown in Figure 4. The process starts with surface sterilization of plant tissues, followed by the isolation of endophytic microorganisms. Various plant parts such as leaves, stems, root can be taken as a source for isolation of endophytes (dos Reis et al., 2022). The preliminary screening of isolates for polysaccharide production can be done based on colony appearance, colorimetric assays like Congo Red Agar (CRA) and Ruthenium Red Agar (RRA), and microscopic examination (Moghannem et al., 2017). Selected EPS-producing strains undergo fermentation in submerged and fed-batch conditions to improve polysaccharide yield (Bakratsas et al., 2024). After cultivation, separation of the fermentation broth from microbial biomass is done by processes such as centrifugation. Pretreatment steps, such as heat treatment or trichloroacetic acid (TCA) deproteinization, are done to remove proteins and impurities (Chen et al., 2012). Next, the crude polysaccharides are recovered through ethanol precipitation, an effective method for isolating EPS (Xu et al., 2020). Then, the precipitated polysaccharides are purified by dialysis to eliminate low-molecular-weight contaminants and then lyophilized to obtain dry polysaccharide fractions.

**Figure 4:**
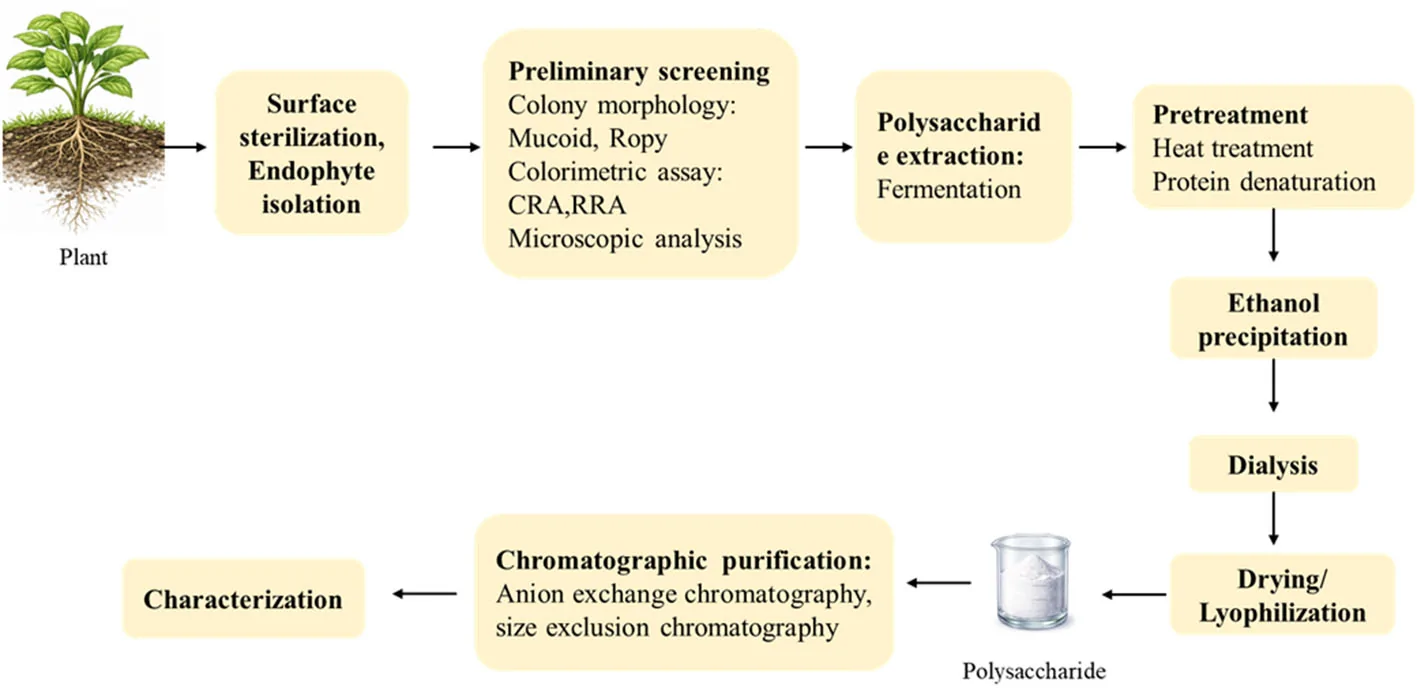
Schematic representation of the isolation, extraction, purification, and structural characterization of exopolysaccharides derived from endophytic microorganisms.

### 6.2 Optimization of fermentation conditions

EPS yield and its structural characteristics are highly dependent on nutrients and physical culture conditions. Optimal tuning of fermentation media is an important but usually underrepresented aspect in EPS production from endophytes. In contrast to free-living environmental bacteria, endophytes are adapted to the highly buffered environment rich in nutrients and chemical signals found within host plant cells. When these endophytes are taken out of this habitat and grown under artificial culture conditions, their productivity, including EPS yield and composition, will largely vary according to how similar the artificial conditions are to those experienced in planta (e.g., *Bacillus rugosus*) (Jaroszuk-Sciseł et al., 2020). Therefore, medium design suitable for one strain of endophytes is not necessarily efficient for another even if both strains are isolated from the same host plant. The optimization can be done using one factor at a time optimization method (OFAT). Physical culture conditions such as inoculation time, temperature, pH of fermentation broth and compositional variations such as Carbon source, nitrogen sources, additional nutrients, salts, etc., can be optimized and can make an optimized media for better production of the EPS. For industrial scale-up, more optimization methods such as RSM can be performed (e.g., EPS production of *Alkalihalobacillus* sp. increased to 3–6 times) (Naykodi et al., 2025).

Glucose is the most frequently reported optimal carbon source for better EPS production from endophytic bacteria and fungi, since it is readily taken up and phosphorylated without the extra catabolic steps required for disaccharides or polysaccharide starting substrates, and it minimizes catabolite repression effects on secondary metabolism (Petry et al., 2000). However, the best carbon source is strain-specific and pathway-specific (e.g., EPS production by *Alkalihalobacillus* sp. is more during glucose supplementation; EPS production by *Bacillus aerophilus* rk1 is more during sucrose supplementation) (Gangalla et al., 2021). Sucrose has been reported to favor mycelial biomass while fructose maximizes EPS yield in some basidiomycetes, and maltose and mannitol were superior to glucose for both biomass and EPS in a survey of eight mushroom species, reflecting differences in sugar-transporter affinity and in the flux of monosaccharides toward nucleotide-sugar precursors used for chain elongation (Ibrahim et al., 2026).

Nitrogen source and C/N ratio regulate the balance between endophyte growth and exopolysaccharide synthesis. Organic nitrogen sources such as yeast extract and peptone often enhance EPS production by supplying amino acids, vitamins, and growth factors; however, excessive nitrogen can favor biomass formation and decrease carbon allocation to EPS. Thus, a relatively high C/N ratio may promote polymer accumulation, although severe nitrogen limitation can also restrict the enzyme synthesis required for EPS biosynthesis (Hereher et al., 2018). Culture growth conditions such as pH and temperature affect enzyme activity, membrane transport, nutrient uptake, EPS secretion, etc. Similarly, aeration influences oxygen availability. Inadequate oxygen limits metabolism, whereas excessive agitation can cause shear stress and result in reduced EPS secretion. Precursors and other elicitors such as salts and mineral ions can enhance EPS production by affecting enzyme activity, membrane permeability, etc. All these parameters are strain-specific. When comparing available data on EPS production optimization, mild stress conditions on the growth of endophytes were found to enhance EPS in more amount (e.g., *Gluconacetobacter diazotrophicus*) (Fu and Yan, 2023; Zhou et al., 2018).

### 6.3 Methods for characterization of EPS

Characterizing endophyte-derived polysaccharides involves determining molecular weight, monosaccharide composition, glycosidic linkages, and overall structure (Figure 5) (Naveen et al., 2023). The structure of exopolysaccharides (EPS) and their functional properties are not fully understood. Because of this, it is important to fully characterize EPS before assessing their biological or physicochemical activities. Key structural parameters that need detailed examination include functional groups, monosaccharide composition, types of sugar residues, ring shapes, molecular weight distribution, branching degree, glycosidic linkage patterns, and surface structure (Table 3) (Zaghloul and Ibrahim, 2022). These features together affect how EPS functions. Many analytical techniques have been used to determine the molecular weight, chemical composition, and structural setup of EPS (Juraskova et al., 2022). However, no one method can fully explain all the structural details of these complex macromolecules. Therefore, a mix of complementary techniques is usually needed for accurate and complete structural characterization (Hakobyan et al., 2023).

**Figure 5:**
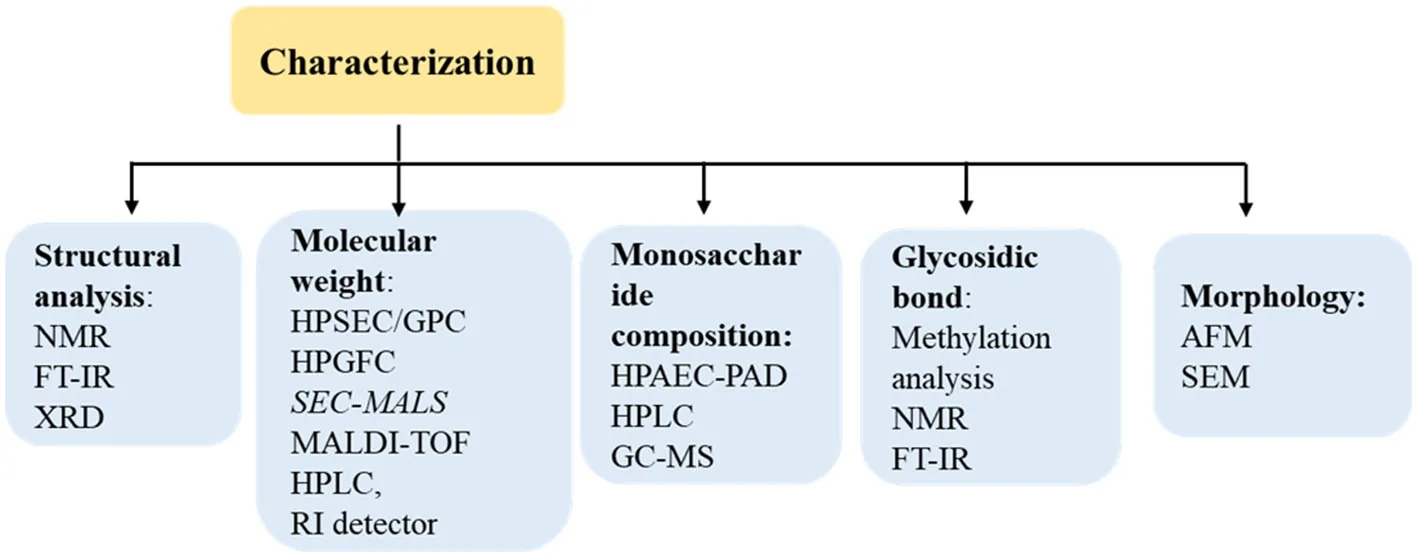
Polysaccharide structural characterization techniques.

**Table 3:** Production, extraction, and characterization methods of polysaccharides from endophytic bacteria and fungi.

No	Endophyte	polysaccharide	Polysaccharide production	Polysaccharide extraction	Molecular weight determination	Monosaccharide composition analysis	Glycosidic linkage analysis	Structural elucidation	References
1	*Burkholderia* sp.	EPS	Fed-batch fermentation (5 L bioreactor)	Fermentation broth separation, ethanol precipitation, and purification	HPSEC/GPC	Acid hydrolysis, chromatographic analysis	Methylation analysis	NMR	Xie et al., 2025
2	*Talaromyces* Sp.	EPS	Submerged fermentation in shake flask	Ethanol precipitation, dialysis, lyophilization	HPGFC	HPAEC	–	FT-IR, NMR	Wang et al., 2025
3	*Ovatospora brasiliensis*	EPS	Fermentation	Dialysis, lyophilization	*SEC-MALS*	HPAEC-PAD, HPLC	–	NMR	Yang et al., 2025
4	*Diaporthe* sp	EPS	Fermentation	Chromatography, lyophilization	HPGPC	HPLC	Methylation and GC–MS analysis, NMR	NMR	Zhang et al., 2025
5	Schizophyllum commune	EPS	Submerge fermentation	Precipitation, lyophilization	HPLC, RI detector	–	–	–	Prathumpai et al., 2025
6	*Ovatospora brasiliensis*	EPS	Fermentation	Ethanol precipitation, dialysis, lyophilization	–	HPLC	NMR, FTIR	NMR, FTIR, XRD	Liang et al., 2025
7	*Aspergillus fumigatus*	EPS	Submerge fermentation	Ethanol precipitation, dialysis, lyophilization	–	HPLC	–	FTIR	Nyaisaba et al., 2025
8	*Preussia isabellae*	EPS	Submerge fermentation	Ethanol precipitation, dialysis, lyophilization	–	HPLC	–	FTIR	Nyaisaba et al., 2025
9	*Methylorubrum*	–	Fermentation	Ion-exchange chromatography followed by gel-filtration chromatography, dialysis, freeze-drying	HPGPC	HPLC	NMR, FTIR	NMR, FTIR, XRD	Fei et al., 2024
10	*Monascus purpureus*	EPS	Fermentation	Ethanol precipitation, dialysis, freeze-drying	HPGPC	HPLC	–	FT-IR, SEM, TGA, DLS	Tao et al., 2024
11	*Penicillium citrinum*	EPS	Fermentation	Ethanol precipitation, dialysis, freeze-drying	HPGPC	HPLC	–	FT-IR, SEM, TGA, DLS	Tao et al., 2024
12	*Aspergillus versicolor*	EPS	Fermentation	Ethanol precipitation, dialysis, freeze-drying	HPGPC	HPLC	–	FT-IR, SEM, TGA, DLS	Tao et al., 2024
13	*Lysinibacillus sphaericus*	EPS	Fermentation	Ethanol precipitation, dialysis, freeze-drying	HPGPC	GC-MS	–	SEM, FT-IR, NMR	Sun et al., 2024
14	*Pseudomonas flavus* Y11	EPS	Submerged fermentation	TCA deproteinization, ethanol precipitation, dialysis, lyophilization	HPGPC	PMP-HPLC	–	UV–Vis, FT-IR, Congo red assay, XRD, SEM, TGA	Long et al., 2024
15	*Rathayibacter oskolensis*	CWPS	Cell wall isolation	Trichloroacetic acid (TCA) extraction, DEAE-cellulose ion-exchange chromatography	Gel permeation chromatography	Acid hydrolysis (2 M HCl, 100 °C, 3 h), Paper chromatography, NMR-based residue identification	2D NMR techniques	NMR, Absolute configuration analysis	Shashkov et al., 2024
16	*Neopestalotiopsis clavispora*	EPS	Submerged fermentation	Ethanol precipitation, dialysis	–	–	–	FT-IR	Koutb et al., 2024
17	*Chaetomium globosum*	EPS	Submerged fermentation	Ethanol precipitation, dialysis, lyophilization	HPGPC	TFA hydrolysis, HPAEC-PAD	–	FT-IR	Wang et al., 2024
18	*Talaromyces purpureogenus*	EPS	fermentation	Ethanol precipitation, dialysis, lyophilization	MALDI-TOF	HPLC	Methylation analysis	FT-IR NMR	Hu et al., 2023
19	*Penicillium janthinellum*	EPS	Fermentation	Ethanol precipitation, dialysis, lyophilization	HPGPC	Reversed-phase HPLC	Methylation analysis	NMR	Shao et al., 2023
20	*Pseudomonas otitidis*	EPS	Fermentation	Ethanol precipitation, dialysis, lyophilization	–	–	–	FT-IR, NMR	Kalimuthu et al., 2023
21	*Bacillus* sp	EPS	Fermentation	Ethanol precipitation, dialysis, lyophilization	HPSEC, MALLS	Acid hydrolysis, HPAEC-IC	–	FT-IR	Zhang et al., 2021
22	Annulohypoxylon thailandicum	EPS	Fermentation	Centrifugation, drying	–	–	–	FT-IR	Yurnaliza et al., 2021
23	*Pilidiella guizhouensis*	EPS	Fermentation	Ethanol precipitation, deproteinization, and lyophilization	HPGPC	GC–MS	–	FT-IR, UV spectroscopy	Zhang et al., 2020
24	*Fusarium solani* DO7	–	Fermentation	Ethanol precipitation, deproteinization and lyophilization, deproteinization, anion-exchange chromatography, gel filtration chromatography	HPGPC	–	Methylation	NMR	Zeng et al., 2019
25	*Alternaria tenuissima*	EPS	Fermentation	Ethanol precipitation, anion-exchange chromatography	HPSEC, MALLS	GC–MS	–	FT-IR, SEM,	Wang et al., 2019
26	*Fusarium* sp. A14	EPS	Fermentation	ethanol precipitation, deproteinization and lyophilization, deproteinization, anion-exchange chromatography, gel filtration chromatography	HPGPC	PMP-HPLC	FT-IR	FT-IR, NMR	Pan et al., 2019

## 7 Molecular weight analysis of EPS

It is a key factor that influences the physical and chemical properties of polysaccharides (Tabani et al., 2025). In materials science, MW impacts tensile strength, elasticity, and biodegradability. Polysaccharides with higher MW are usually more rigid and stable, making them ideal for structural and packaging applications (Fan et al., 2014). In biomedical fields, high- MW polysaccharides (over 100 kDa) can create organized structures like triple helices. These structures enhance immunomodulatory and antitumor activities by interacting with receptors (Xu et al., 2022). In food systems, MW affects solubility, viscosity, and fermentability. These factors affect texture, fullness, and prebiotic potential since lower MW substances are usually more easily fermented by gut bacteria (Canazza et al., 2025). Therefore, determining MW is important for optimizing the use of polysaccharides in industrial, nutritional, and therapeutic applications. Various analytical techniques measure the molecular weight of polysaccharides, including chromatographic and mass spectrometric methods (Yadav et al., 2024). The most commonly used methods are described below.

### 7.1 Size exclusion chromatography (SEC)

SEC can be used for the analysis of the molecular weight of polysaccharides. Molecules are separated according to their size and hydrodynamic volume (La Verde et al., 2017; Schure and Moran, 2017). In this method, molecules are separated as they move through a porous stationary phase, with larger molecules unable to enter the pores and being eluted first, while smaller molecules can access the pores to different extents and are eluted afterwards. The high-performance variant, High-performance size exclusion chromatography (HPSEC), enables precise determination of key molecular weight parameters including number-average molecular weight (Mn), weight-average molecular weight (Mw), and polydispersity index (PDI) (White et al., 1999). This method is non-destructive, quick, and only needs minimal sample preparation, which makes it especially useful for characterizing polysaccharides (Zhou et al., 2000; Boysen and Hearn, 2010).

HPLC with a Refractive Index (RI) detector is a main, cost-effective, and universal method for measuring the molecular weight (MW) distribution of polymers, carbohydrates, and alcohols that do not absorb UV light (Boysen and Hearn, 2010). In SEC-HPLC, separation relies on hydrodynamic volume instead of chemical interactions. Larger molecules cannot penetrate the porous stationary phase and therefore exit the column more quickly. In contrast, smaller molecules are able to access the pores, resulting in a later elution. A calibration curve of log molecular weight vs. elution volume is created by using standards with known molecular weights. This procedure allows for the estimation of the number-average molecular weight (MW) as well as the polydispersity index (MW/MN) of unknown polysaccharides. Hydrophilic polymeric SEC columns are often used with aqueous mobile phases to analyze water-soluble polysaccharides, including amylose, amylopectin, xanthan gum, pullulan, guar gum, and carboxymethyl cellulose (Reuhs and Rounds, 2010; Mhatre and Krull, 1993).

RI detector provides relative molecular weights, and it is sensitive to temperature changes. But it is a reliable method for polymer characterization. For more accurate and absolute determination of molecular weight, SEC can be paired with light scattering detectors like low-angle laser light scattering (LALLS) or multi-angle laser light scattering (MALLS) (Grcev et al., 2004). These detectors do not require calibration standards and allow direct measurement of molecular weight and molecular size.

### 7.2 SEC coupled with multi-angle light scattering (SEC-MALS/MALLS)

Static light scattering (SLS) is a reliable and easily accessible method for calculating the absolute molecular weights of polymers. This method can be combined with in-line multi-angle light scattering (MALS) detectors and integrated into SEC. Now, this SEC-MALS has become a potent tool for accurately characterizing the absolute molecular weight of proteins and polymers (Matson et al., 2024). Compared with other methods, this SEC-MALLS provides molecular weight data directly (Thomsen, 2020).

### 7.3 Matrix-assisted laser desorption/ionization time-of-flight mass spectrometry (MALDI-TOF-MS)

MALDI-TOF mass spectrometry is a soft-ionization technique used to determine the molecular weight of various biomolecules, including proteins, peptides, glycans, lipids, synthetic polymers, and polysaccharides (Fatema et al., 2010). It is especially useful for analyzing polysaccharides and their oligosaccharide fragments. This method provides precise molecular mass data with little fragmentation and fast analysis. In the analysis, the sample is mixed with a suitable substance, such as 2,5-dihydroxybenzoic acid, and co-crystallized onto a target plate. When a laser hits the sample, the matrix absorbs energy and facilitates the ionization of the analyte while minimizing fragmentation. The ions generated are propelled through a vacuum tube, and their time of flight is measured. Because lighter ions travel faster than heavier ones, the flight time corresponds directly to the mass-to-charge ratio (m/z). By calculating m/z values from the recorded flight times, we can determine the molecular weight of the analyte (Sebela, 2022). MALDI-TOF offers high speed, good sensitivity, little sample preparation, and tolerance to impurities. This makes it especially suitable for quickly determining the molecular weight of low- to medium-molecular-weight polysaccharides and their oligosaccharide fragments (Li et al., 2022).

## 8 Monosaccharide composition analysis

The composition of monosaccharides is an important feature affecting the biological activities of EPSs (Wei et al., 2023). A comparative analysis of the data in Tables 1, 2 shows clear structural differences between bacterial and fungal endophytic polysaccharides in terms of monosaccharide composition, glycosidic linkages, molecular weight distribution, and related biological activities. Polysaccharides from bacterial endophytes are mainly heteropolysaccharides composed of various sugar residues, including glucose, mannose, galactose, rhamnose, arabinose, xylose, fucose and often uronic acids like glucuronic acids and galacturonic acids (Table 1). Many bacterial EPSs are rich in mannose or contain significant amounts of charged uronic acids. These components improve solubility, viscosity, emulsifying abilities, and antimicrobial effects. Structurally, these endophytic bacterial polysaccharides have complex repeating units with mixed α and β glycosidic linkages and branches. Endophytic fungal polysaccharides have glucose as the major monosaccharide, but some fungal polysaccharides also contain mannose, galactose, rhamnose, fucose, and uronic acids. Fungal polysaccharides show a broader molecular weight distribution, ranging from low kilodaltons, depending on the strain (Table 2). Higher molecular weight β-glucans are typically linked to immune-modulating, anti-inflammatory, antitumor, wound-healing, and liver-protecting activities. These structural characteristics of polysaccharides can be determined using various analytical techniques such as HPAEC-PAD, HPLC, GC-MS, etc.

### 8.1 High-performance anion-exchange chromatography with pulsed amperometric detection (HPAEC-PAD)

HPAEC-PAD is a popular method for analyzing glycoproteins and carbohydrates. It was first reported by (Hardy et al., 1988) that HPAEC-PAD could be used to determine the monosaccharide content of a glycoprotein (Hardy et al., 1988). For monosaccharide composition analysis, the glycoproteins or other glycoconjugates will be subjected to acid hydrolysis using a volatile acid like trifluoroacetic acid (TFA). This process breaks the glycosidic linkages and releases individual monosaccharides. The dried residue sample is then reconstituted in deionized water and can be introduced into HPAEC-PAD. The separation of individual monosaccharides can be achieved by a Dionex CarboPac PA1 column using an isocratic mobile phase (e.g., 16 mM sodium hydroxide). The identification of monosaccharides can be achieved through pulsed amperometric detection, which determines the current from the oxidation of carbohydrates at a gold electrode. This method enables sensitive and direct quantification without any prior derivatization (Rohrer et al., 2013).

### 8.2 High-performance liquid chromatography (HPLC)

HPLC can be used to detect monosaccharide composition (Dey et al., 2023). Before analysis, the polysaccharide undergoes acid hydrolysis to release its monosaccharides. Typically, the purified polysaccharide is treated with trifluoroacetic acid (TFA), which breaks glycosidic linkages and converts the polysaccharide into free monosaccharides (Shi et al., 2020). The prepared sample is injected into an HPLC system with suitable detectors, such as a refractive index (RI) detector. Separation happens on columns like the Rezex ROA-Organic Acid H^+^ column using an isocratic mobile phase, at a controlled flow rate and temperature. Individual monosaccharides are identified and measured by comparing them with external standards using calibration curves. This process allows for a precise determination of the EPS monosaccharide profile (La Torre et al., 2024).

### 8.3 Gas chromatography-mass spectrometry (GC-MS)

This technique can be used to identify the monosaccharide composition and glycosidic linkages of polysaccharides (Zhu et al., 2024). The monosaccharide composition analysis involves three key steps. First, the carbohydrate undergoes quantitative solvolysis through acid hydrolysis or methanolysis. This process breaks glycosidic bonds and releases individual monosaccharide components. Secondly, because monosaccharides are non-volatile and thermally unstable, they are chemically modified, commonly by trimethylsilylation, to change hydroxyl groups into volatile trimethylsilyl (TMS) derivatives. Third, these TMS derivatives are separated on a GC column and measured based on their retention times and peak areas, usually using flame ionization detection (FID) or mass spectrometry (MS) (Sato, 2021).

## 9 Structural analysis of polysaccharides

### 9.1 Fourier transform infrared (FT-IR) spectroscopy

It is used to identify the functional groups that are present in the EPS (Wei et al., 2023). By analyzing the absorption of infrared light, FT-IR give peak highlights of specific chemical bonds and functional groups. The characteristic functional group stretching vibrations in polysaccharides include O–H stretch (3,200–3,500 cm^−1^), C–H stretch (around 2,900 cm^−1^), C=O stretch of carboxyl/acetyl groups (around 1,720–1,740 cm^−1^), asymmetric and symmetric COO^−^ stretches (around 1,600 and 1,400 cm^−1^), and C–O–C glycosidic linkage vibrations (around 1,000–1,150 cm^−1^) (Szymanska-Chargot and Zdunek, 2013). These collectively provide a functional group fingerprint that helps with polysaccharide identification and structural characterization. The FT-IR analysis of the sample can be done in two major ways. One is Attenuated Total Reflectance (ATR), where both liquid and dry powder samples can be analyzed, and this method doesn't require much sample preparation time, so results can be obtained quickly (Van Haaren et al., 2023). In this method, an ATR crystal with a high refractive index such as diamond, zinc selenide (ZnSe), germanium (Ge), and silicon (Si), etc., acts as the sample support (Etemadi et al., 2025). Infrared radiation undergoes total internal reflection within that crystal and generates an evanescent wave that penetrates the sample surface when the incident angle exceeds the critical angle (Burgi, 2011).

Another method involves the KBr pellet method, where the sample is finely ground and mixed with spectroscopic grade potassium bromide (KBr), usually in a 1:100 ratio. This homogeneous mixture is then compressed into a transparent pellet using a hydraulic press (Hong et al., 2021). The sample will be analyzed. The obtained peak of the functional group is interpreted for results.

### 9.2 Nuclear magnetic resonance (NMR)

This spectroscopy technique is for the structural characterization of exopolysaccharides (EPS). Purified EPS (e.g., 50 mg) is dissolved in deuterium oxide (D_2_O) to eliminate interference from exchangeable protons and subjected to detailed spectroscopic analysis. Both one-dimensional (^1^H and ^13^C NMR) and two-dimensional (2D) NMR are used to obtain comprehensive structural information (Liu et al., 2024). One-dimensional (1D) NMR spectroscopy gives important structural information by showing chemical shifts of nuclei in one dimension. This makes it helpful for basic molecular identification and checking purity. On the other hand, two-dimensional (2D) NMR spectroscopy addresses the limits of 1D NMR. It reveals connections between nuclei using methods like COSY, HSQC, and HMBC. This allows for a detailed understanding of complex molecules in pharmaceutical research and drug development (Ahmed, 2024). The ^1^H-NMR (Proton NMR) spectrum displays the chemical shifts associated with various types of protons present in the sample. On the other hand, ^13^C NMR provides structural information about a compound based on the chemical shifts of various types of carbon (Gunawan and Nandiyanto, 2021). Overall, NMR spectroscopy is a strong, non-destructive method commonly used to identify the monosaccharide composition, anomeric configuration, glycosidic linkages, and overall structure of exopolysaccharides (EPS) (Fontana and Widmalm, 2023).

### 9.3 X-ray crystallography (XRD)

This technique is used to determine the crystallographic characteristics of materials by analyzing the patterns of diffraction produced by the interaction of X-rays and the crystalline specimens. This analysis gives useful information such as lattice parameters, structural configuration, and crystal alignment, which helps to optimize material synthesis (Aljawrneh et al., 2025). The fundamental principle of XRD involves the interaction of X-rays with the atomic arrangement in crystalline materials.

When X-rays strike a crystalline material, they are elastically scattered by the atomic planes, producing diffraction patterns according to Bragg's law. Since the wavelength of X-rays (about 0.15–0.5 nm) is comparable to the distance between atoms in crystals, constructive and destructive interference occurs, generating characteristic diffraction peaks. XRD analysis involves mounting a purified sample on the sample holder and scanning over a 2θ range of 5–90° at a controlled scanning speed to record the diffraction pattern. The diffractogram obtained makes it possible to determine the degrees of crystallinity and structural organization of the polysaccharide. In many studies, microbial polysaccharides typically exhibit broad diffraction peaks in the XRD pattern. This suggests that the polysaccharide is mainly amorphous rather than crystalline (Yadav et al., 2024).

## 10 Structure- activity relationship (SAR) of endophyte-derived polysaccharides

The structural features of endophyte-derived exopolysaccharides are highly significant determinants of their biological activity (Table 4). Rather than carbohydrate composition alone, factors such as molecular weight, monosaccharide composition, glycosidic linkage patterns, branching, and functional group modifications collectively influence their functional properties. Understanding these structure-activity relationships can reveal why exopolysaccharides with similar chemical compositions show significant differences in their biological activities. Based on the composition, molecular-weight, and glycosidic linkage data summarized in Tables 1, 2, the following subsections discuss the major structural features influencing the biological activities of polysaccharides derived from endophytes. Where endophyte-specific evidence is limited, relevant findings from the broader microbial polysaccharide literature are included to support the discussion.

**Table 4:** Summary table of structure-activity relationship.

Structural feature	Category/range	Examples	Associated bioactivity	Proposed mechanism
Molecular weight	High (>100 kDa)	*Ovatospora brasiliensis, Schizophyllum commune, Diaporthe* sp.	Immunomodulatory, antitumoral, anti-inflammatory, moisture-retentive, hepatoprotective	Forms ordered secondary structures (single/triple helices) that interact with immune pattern-recognition receptors
	Low (< 10 kDa)	*Talaromyces purpureogenus, Fusarium solani DO7 and Penicillium janthinellum*	Antioxidant, wound-healing, antibacterial, anti-diabetic	More reducing ends/hydroxyl groups per unit weight (better radical scavenging); small size aids membrane diffusion
Intermediate (10–50 kDa)	*Bacillus* sp.*, Alternaria tenuissima*	Moderate antioxidant, antitumor and antibacterial activity	Intermediate structural flexibility and reactivity
Monosaccharide composition	High uronic acid content (glucuronic/galacturonic acid)	*Monascus purpureus, Penicillium citrinum, Aspergillus* sp.*, Pseudomonas flavus Y11*	Strong antioxidant activity	Carboxyl groups enhance electron donation and metal-ion chelation, improving radical scavenging
Glycosidic linkage pattern	β-1,3-glucan backbone	*Talaromyces* sp.	Anti-inflammatory/immunomodulatory	β-glucan structure engages innate immune pattern-recognition receptors
	1,4 and 1,6-glucopyranosyl linkages	*Diaporthe* sp.	Moisture-retentive, hepatoprotective	Linkage position alters function even with the same monosaccharide (glucose)
Anomeric configuration	β-anomeric (β-1,3, β-1,6)	*Talaromyces* sp*., Schizophyllum commune*	Anti-inflammatory, immunostimulatory, antitumor	Recognized by receptors like dectin-1, complement receptor 3, TLRs
	α-anomeric	*Burkholderia* sp.*, Fusarium* sp. *A14*	Antioxidant, prebiotic, antibacterial	Not recognized by immune pattern-recognition receptors
	Mixed α/β	*Fusarium solani DO7*	Broader, multi-pathway bioactivity	May engage multiple biological pathways simultaneously, possibly at reduced potency per pathway

### 10.1 Molecular weight and bioactivity

Molecular weight (MW) is a major determinant of polysaccharide activity. But its relationship with biological activity is not linear. Polysaccharides with high molecular weights (greater than 100 kDa), like the EPS from *Ovatospora brasiliensis* (100 to 1,000 kDa) and *Schizophyllum commune* (5,000 kDa in some strains), tend to exhibit an ordered secondary structure such as single or triple helices when dissolved in water. The ordered secondary structures of these polysaccharides are necessary for interaction with pattern recognition receptors present on immune cells and are responsible for their immunomodulatory, antitumoral, and anti-inflammatory properties (Batbayar et al., 2012). Extremely high molecular weight makes the chains entangled, a property that is favorable for gel formation and moisture retention, as can be observed in *Diaporthe* sp. EPS (462 kDa; moisture-retentive, hepatoprotective).

Some of the exopolysaccharides with powerful antioxidant and wound-healing potential, listed in Table 2, are low molecular weight compounds; for example, 5 kDa EPS of *Talaromyces purpureogenus* has both antioxidant and wound-healing potential. 1.3–10 kDa fractions of *Fusarium solani DO7* and *Penicillium janthinellum* have antibacterial and anti-diabetes activity, respectively. Low molecular weight polysaccharides exhibit more reducing ends and hydroxyl groups in the molecule per unit weight, thus demonstrating greater efficiency in free radical scavenging; due to their small size, these compounds diffuse more easily through biological membranes and barriers, making them effective in wound healing and antidiabetic properties (Chen et al., 2013). Intermediate molecular weight polysaccharides with approximately 10–50 kDa, such as EPS of *Bacillus* sp. (18.63 kDa) and *Alternaria tenuissima* EPS (32.46 kDa), typically possess intermediate antioxidant properties along with antitumor or antibacterial action.

### 10.2 Monosaccharide composition and specific activities

The monosaccharide composition of exopolysaccharides is a major determinant of its physiochemical characteristics and biological activities. This influences properties such as molecular conformation, solubility, charge distribution, and interaction with biological targets, etc. (Fan et al., 2026).

The polysaccharides with more uronic acids, such as glucuronic acids and galacturonic acids, are more associated with strong antioxidant activity. Many antioxidant-active polysaccharides listed in the tables, including EPS from *Monascus purpureus, Penicillium citrinum, Aspergillus and Pseudomonas flavus* Y11, contain appreciable proportions of uronic acids. The presence of carboxyl groups in these residues enhances the electron donation ability and facilitates transition metal ion chelation. This further improves hydroxyl radical and superoxide anion scavenging activity (Chen et al., 2024).

### 10.3 Glycosidic linkage patterns and biological function

The glycosidic linkage pattern is another important structural feature influencing the biological activity of polysaccharides. When analyzing the reported data in Tables 1, 2, β-1,3 glucan backbones are consistently associated with immunomodulatory functions. The EPS from *Talaromyces* sp., characterized by predominant β-1,3-D-glucopyranosidic linkages, exhibited anti-inflammatory activity, supporting the well-established role of β-glucans in modulating innate immune responses through pattern-recognition receptors. In contrast, the EPS from *Diaporthe* sp., containing 1,4- and 1,6-glucopyranosyl linkages, demonstrated moisture-retentive and hepatoprotective properties, suggesting that changes in linkage position can substantially alter the functional behavior of polysaccharides even when glucose remains the principal monosaccharide (Zhong et al., 2023). Compared to the β-linkages, EPS with more ∝-linkages do not exhibit a clear association with a single biological function. Even though more data are needed for a conclusion, the existing data reveal that bioactivity cannot be attributed solely to anomeric configuration. But it is likely influenced by combined effects of monosaccharide composition, molecular weight, branching pattern, and higher-order structure.

### 10.4 Anomeric configuration (α vs. β) and receptor interaction

The anomeric configuration is a crucial characteristic defining which biological receptors a polysaccharide can interact with. β-anomeric glucans, particularly β-1,3 and β-1,6 -linked chains, are specifically recognized by innate-immune pattern-recognition receptors such as dectin-1, complement receptor 3, and select toll-like receptors, etc., which explains why the β-glucan-rich EPS in Table 2 (e.g., *Talaromyces* sp. and *Schizophyllum commune*) are disproportionately associated with anti-inflammatory, immunostimulatory, and antitumor activity (Elder et al., 2017). On the other hand, ∝-anomeric configurations, as seen in EPS of bacterial species *Burkholderia* sp. and fungal species *Fusarium* sp. *A14* are generally not recognized by the same receptors. They are reported with activities such as antioxidant, prebiotic, or antibacterial activities that do not require immune-receptor engagement. When ∝ and β linkages co-occur within a single heteropolysaccharide (e.g., EPS of *Fusarium solani DO7*), the reported bioactivity profile is often broader. This suggests that the mixed anomeric configuration may allow a single polysaccharide to engage more than one biological pathway, at the probable cost of maximal potency in either.

## 11 Current limitations and future perspectives

Despite the significant advances in research on endophyte-derived polysaccharides, several limitations remain. A major challenge is the accurate isolation and identification of true endophytes. Because microorganisms can associate themselves with senescent or dead parts of plants as epiphytes or saprophytes, it is difficult to distinguish between true endophytes and external colonizers. Additionally, most existing research work covers only a limited number of culturable isolates obtained from a single host species or location, which may not adequately represent the true diversity of polysaccharide-producing endophytes. Many endophytic microorganisms remain unculturable under conventional laboratory isolation conditions, leaving a significant fraction of their biosynthetic potential unexplored. Despite many reported studies showing promising biological activities, the detailed structural characterization is often incomplete, with limited information about their glycosidic linkage, branching architecture, molecular conformation, and structure- activity relationship. The absence of standardized methods for plant surface sterilization, endophyte isolation, polysaccharide extraction, purification and characterization limits reproducibility and comparisons between studies. In addition, most research work remains confined to *in vitro* assays, whereas mechanistic studies, toxicity evaluation, *in vivo* validation, and scalable production strategies are still scarce.

## 12 Conclusion

Endophytic microorganisms have been recognized as important sources of structurally varied polysaccharides, especially exopolysaccharides (EPS), which hold potential for use in the pharmaceutical, biomedical, agricultural and industrial fields. This review encapsulates the existing knowledge on polysaccharides sourced from both fungal and bacterial endophytes, emphasizing that fungal endophytes have been the most thoroughly studied as producers of EPS. However, growing evidence suggests that bacterial endophytes also constitute a valuable and largely untapped source of polysaccharides.

The key emphasis of this review is the thorough compilation of techniques used for polysaccharide characterization, encompassing production methods, extraction and purification practices, molecular weight assessment, analysis of monosaccharide composition, examination of glycosidic linkages, and sophisticated structural characterization approaches. Overall, this review serves as a comprehensive resource for understanding various endophyte-derived polysaccharides and characterization techniques. Collectively, the information presented provides a consolidated overview of the field and highlights the methodological advances that have contributed to the characterization of endophyte-derived polysaccharides.
